# Modeling surgeon belief updating under bias: a Bayesian simulation in shoulder arthroplasty

**DOI:** 10.1016/j.jseint.2025.09.018

**Published:** 2025-10-24

**Authors:** Mariano E. Menendez, Michael A. Moverman, Surena Namdari, Frederick A. Matsen, David Ring

**Affiliations:** a Department of Orthopaedic Surgery, University of California Davis, Sacramento, CA, USA; b Department of Orthopaedic Surgery, Duke University, Durham, NC, USA; c Rothman Orthopaedic Institute, Thomas Jefferson University, Philadelphia, PA, USA; d Department of Orthopaedics and Sports Medicine, University of Washington, Seattle, WA, USA; e Department of Surgery and Perioperative Care, Dell Medical School at the University of Texas at Austin, Austin, TX, USA

**Keywords:** Bayesian simulation, Belief updating, Confirmation bias, Surgeon decision-making, Practice variation, Shoulder arthroplasty

## Abstract

**Background:**

Shoulder surgeons often offer markedly different treatment recommendations for a given patient and pathophysiology within the context of a common body of evidence. Prior beliefs and cognitive bias may contribute to this variability. Using Bayesian decision theory informed by principles of behavioral science, we modeled how surgeons with different initial beliefs and degrees of bias update their treatment preferences in response to new evidence comparing anatomic total shoulder arthroplasty (aTSA) and reverse total shoulder arthroplasty (rTSA) when making surgery recommendations for advanced primary glenohumeral osteoarthritis.

**Methods:**

We developed a Bayesian simulation involving 3 hypothetical surgeons with distinct initial beliefs: aTSA Loyalist (90% belief aTSA is superior), Neutral Thinker (50%), and rTSA Advocate (10% belief aTSA is superior) and varying degrees of confirmation bias (eg, the selective discounting of evidence that contradicts one's current belief). Each surgeon was sequentially exposed to 10 simulated randomized trials modestly favoring rTSA, with belief trajectories updated after each trial under 2 conditions: (1) an unbiased scenario, in which all new evidence was weighted at face value, and (2) a biased scenario, in which disconfirming evidence was systematically downweighted.

**Results:**

Under a condition simulating no confirmation bias, all surgeons gradually shifted toward lower belief in aTSA superiority as they reviewed the 10 rTSA-favoring trials: the aTSA Loyalist moved from about 90% to 25% confidence in aTSA, the Neutral Thinker from 50% to 2.0%, and the rTSA Advocate from 10% to 1.2%. Under biased conditions, belief change was markedly reduced for the aTSA Loyalist, who remained 64% confident in aTSA superiority despite consistent rTSA-favoring evidence. Changes for the rTSA Advocate (10%-1.2%) and Neutral Thinker (50%-2.3%) were largely unchanged.

**Conclusion:**

This Bayesian simulation provides a practical framework to demonstrate how prior beliefs and cognitive bias can markedly influence the way shoulder surgeons interpret and act upon new evidence, contributing to unwarranted variation in care. When it comes to treatment recommendations, what surgeons believe at the outset may matter as much or more than the data itself. Implementing targeted strategies such as foundational principles based in behavioral ethics, evidence-based decision and debiasing aids, structured peer review, and routine performance feedback may help align treatment decisions more closely with a patient's values based on the best available evidence. The use of rTSA-favoring evidence in this simulation is solely for illustrative purposes and should not be interpreted as an endorsement of increased rTSA use in clinical practice.

Surgeons faced with the same clinical scenario may choose markedly different operative strategies. In shoulder surgery, the choice between anatomic total shoulder arthroplasty (aTSA) and reverse total shoulder arthroplasty (rTSA) for primary glenohumeral osteoarthritis illustrates this phenomenon.^6^ Despite a growing observational and registry evidence base,^2^^,^^4^^,^^7^ uncertainty persists, and practice patterns remain highly variable.One potential explanation for this variability lies in how surgeons integrate new evidence into pre-existing mental frameworks. Bayesian decision theory is a mathematical framework that provides a normative model for belief updating: prior probability estimates are modified in proportion to the strength and direction of new data, yielding a posterior probability that guides future decisions.^8^ In clinical practice, however, cognitive and contextual factors frequently distort this process. Surgeons may exhibit confirmation bias (eg, the selective discounting of evidence that contradicts one's current belief) by applying asymmetric skepticism, in which disconfirming data are scrutinized more critically than evidence that supports their preferences.^1^ Anchoring to early-career experiences, loyalty to a mentor's teaching, and a professional identity tied to a specific technique can further reinforce this belief inertia. As a result, identical streams of new evidence can produce markedly different belief trajectories among surgeons. In some cases, even consistent and credible data may fail to meaningfully shift practice if prior beliefs are strong and confirmation bias is not ameliorated. This dynamic is rarely quantified in surgery, despite its likely contribution to unwarranted variation in care.

In this study, we developed a Bayesian decision theory model to examine how surgeons with different initial beliefs and degrees of bias update their treatment preferences in response to new evidence comparing aTSA and rTSA for primary glenohumeral osteoarthritis. The purpose is not to adjudicate the optimal arthroplasty choice for shoulder osteoarthritis but rather to illustrate the degree to which prior beliefs and cognitive bias can shape the assimilation of evidence in surgical decision-making. This study introduces a practical framework that conceptualizes surgical practice variation as a dynamic process of belief updating under bias.

## Methods

We developed a Bayesian decision theory model to examine how prior beliefs and cognitive bias influence the assimilation of new evidence in surgical decision-making. In simple terms, Bayesian theory means starting with a probability based on prior knowledge, then adjusting that probability step by step as new information becomes available, much like updating one's opinion as additional pieces of evidence are revealed. The model compared belief updating under 2 conditions: (1) unbiased Bayesian updating, in which all new evidence is weighted proportionally to its strength, and (2) biased updating, in which evidence contradicting the initial belief was selectively discounted or disregarded, simulating the effects of confirmation bias. The clinical context was the choice between aTSA and rTSA for primary glenohumeral osteoarthritis. This simulation was designed to illustrate belief dynamics and cannot address the relative benefits and harms of the 2 procedures.

Three hypothetical surgeons were defined according to their initial belief (prior probability) that aTSA was superior to rTSA: (1) aTSA Loyalist = 0.90 (eg, 90% belief aTSA is superior); (2) Neutral Thinker = 0.50; (3) rTSA Advocate = 0.10 (eg, 10% belief aTSA is superior). These values were selected to represent a wide range of pre-existing attitudes, from strong preference for aTSA, to equipoise to strong preference for rTSA. The exact percentages were chosen for conceptual clarity and to produce visually distinct belief trajectories.

We simulated 10 sequential randomized controlled trials comparing aTSA and rTSA. Each trial was designed to represent a modest and consistent advantage for rTSA, chosen for illustrative purposes only. Effect sizes were held constant across all trials to isolate the influence of prior beliefs and confirmation bias on belief updating rather than trial-to-trial variability. All surgeons received the same sequence of trial results in the same order.

We modeled belief change using the principles of Bayes' theorem. In practical terms, this means that a surgeon's starting belief about which procedure is better (the *prior*) is adjusted after reviewing each new trial (the *evidence*), resulting in an updated belief (the *posterior*). The updated belief from one trial then becomes the starting point for the next trial. If the new evidence supports the surgeon's current belief, the probability that they are correct increases. If the new evidence contradicts their belief, that probability decreases. The size of the change depends on both the strength of the evidence and how strongly the surgeon held their belief to begin with. In the unbiased scenario, all evidence was weighted equally. In the biased scenario, disconfirming evidence was given less weight before updating, simulating the effect of confirmation bias.

The updating process can be expressed in odds form as:

PosteriorOdds=PriorOddsxLikelihoodRatio

Where:

$$Prior\;Odds=\frac{P\;prior}{1-P\;prior}$$

$$Likelihood\;Ratio=\frac{Probability\;of\;the\;evidence\;if\;TSA\;is\;superior}{Probability\;of\;the\;evidence\;if\;RSA\;is\;superior}$$

$$Posterior\;Probability=\frac{Posterior\;Odds}{1+Posterior\;Odds}$$

In the biased scenario, confirmation bias was modeled by reducing the impact of disconfirming evidence through a bias factor *w*:

PosteriorOdds=PriorOddsx(LikelihoodRatio)w

Where:

*w* = 1.0 in the unbiased scenario (no discounting).

*w* = 0.5 for disconfirming evidence in the biased scenario.

*w* = 1.0 for confirming evidence in both scenarios.

The bias factor of 0.5 was selected to represent a moderate degree of confirmation bias, consistent with prior behavioral science modeling studies,^3^ and was chosen to yield visually distinguishable biased and unbiased belief trajectories without eliminating all belief change. Calibration was held constant to isolate belief dynamics rather than variable study quality.

The primary outcome was the posterior probability that aTSA was superior to rTSA after each trial. Belief trajectories for each surgeon profile were plotted for both unbiased and biased conditions. All simulations were conducted in Python version 3.11 (Python Software Foundation, Wilmington, DE, USA) using NumPy for probabilistic calculations and Matplotlib for data visualization.

## Results

When all surgeons updated their beliefs without bias, trajectories converged toward the true underlying effect. The aTSA Loyalist, who began with a 90% probability that aTSA was superior, demonstrated a steady decline in belief across the 10 trials, ultimately reaching approximately 25%. The Neutral Thinker, starting at 50%, decreased to 2.0% by the end of the sequence. The rTSA Advocate, whose initial belief in aTSA superiority was only 10%, declined further to about 1.2%. This pattern illustrates the expected behavior under unbiased Bayesian updating, where repeated exposure to consistent rTSA-favoring evidence gradually shifts beliefs in the same direction for all surgeons, regardless of their starting point.

Introducing confirmation bias markedly altered the belief trajectory for the aTSA Loyalist. Because all 10 trials favored rTSA, the aTSA Loyalist applied the bias factor to each trial, slowing the rate of belief change and resulting in a final probability of 64%—a 39-percentage-point difference compared with the unbiased scenario. For the Neutral Thinker, the first rTSA-favoring trial shifted belief below the 50% threshold, making all subsequent evidence confirming rather than disconfirming; therefore, the bias factor was not applied, and the trajectory (50%-2.3%) was nearly identical to the unbiased scenario. For the rTSA Advocate, all evidence supported the preferred procedure, so bias weighting had no effect; the final probability was 1.2% in both scenarios.

**Figure 1:**
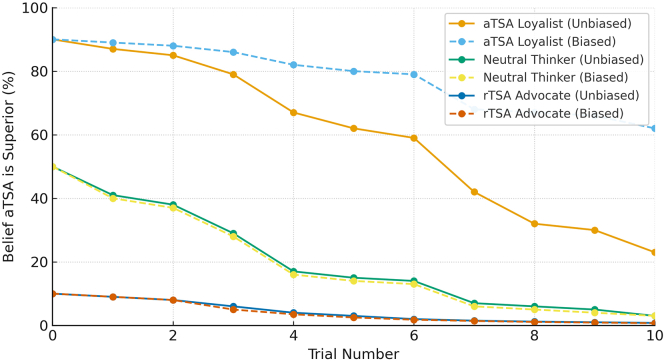
Comparison of biased vs. unbiased belief updating for all 3 surgeons. *aTSA,* anatomic total shoulder arthroplasty; *rTSA*, reverse total shoulder arthroplasty.

**Figure 2:**
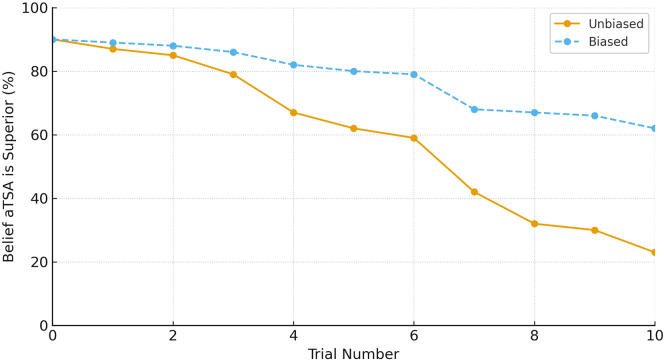
Focused comparison of biased vs. unbiased belief updating on the aTSA Loyalist. *aTSA*, anatomic total shoulder arthroplasty.

Figure 1 displays the direct comparison of biased vs. unbiased updating for all 3 surgeons. Figure 2 focuses on the aTSA Loyalist as a case example, illustrating the substantial persistence of a strong prior in the presence of confirmation bias.

## Discussion

This Bayesian simulation demonstrates how prior beliefs and confirmation bias can markedly influence the assimilation of new evidence in surgical decision-making. In other words, our study highlights that, when it comes to evidence, what surgeons believe at the outset may matter as much or more than the data itself. Even when evidence is consistent, unbiased in generation, and of uniform quality, belief trajectories diverge substantially depending on both the starting point and the weighting applied to disconfirming information. This suggests that persistent variation in practice patterns may not solely stem from gaps in the evidence base, but from differences in how individual surgeons interpret and integrate the same data. By linking practice variation to underlying belief dynamics, this work offers a practical, easily transferable framework for studying decision-making in other surgical domains. Variation in surgical treatment is not inherently problematic when outcomes are equivalent; the concern arises when differences in practice reflect entrenched bias rather than evidence aligned with patient outcomes and values.

The aTSA Loyalist archetype illustrates an important point: strong priors are not necessarily irrational—they may reflect years of training, personal experience, and patient outcomes—but they can act as a cognitive filter that reduces the perceived credibility or relevance of conflicting findings. The Neutral Thinker's modest bias effect suggests that surgeons with balanced prior beliefs tend to respond more readily to new evidence, while the rTSA Advocate's minimal change shows another face of bias—a steady belief when new data reinforces existing views. There is no correct mindset, but it is important for each surgeon to become aware of where they stand on a matter of debate, anticipate cognitive biases such as anchoring, framing, confirmation, and self-serving biases, and be prepared with cognitive debiasing tactics in order to be more adaptive to quality evidence.

Beyond the immediate scope of comparing aTSA and rTSA, our findings underscore the notion that efforts to limit variations in care to those that reflect what matters most to the patient—unclouded by misconceptions and distress—and also address effective use of resources cannot rely solely on generating more data but instead on addressing how surgeons engage with and internalize that data. For instance, shoulder surgeons with strong pre existing treatment preferences may require more than a few high-quality experiments to reconsider their practice. Structured peer-to-peer discussions, exposure to contrasting operative approaches through visiting professorships or live surgical observation, and deliberate reflection on registry-derived outcomes may provide constructive opportunities to challenge and refine entrenched prior beliefs.

From a systems perspective, recognizing that belief dynamics influence surgical decision-making creates an opportunity for targeted interventions. Clinical guidelines, decision aids, and registry feedback can be designed with an awareness of cognitive biases, framing evidence in ways that both affirm the clinician's expertise and promote openness to alternative approaches.^5^ Multidisciplinary case conferences that explicitly surface and discuss differing priors can make variation visible and foster more balanced deliberation. By treating belief updating as a modifiable process rather than a fixed trait, surgical communities can foster a culture where evidence has a clearer path to influencing practice, ultimately reducing unwarranted variation and aligning treatment decisions more closely with what matters most to patients. Recognizing the role of belief dynamics provides a deeper lens for understanding why treatment consensus is often elusive while offering concrete opportunities to narrow the gap between what the data suggest and what patients are offered. The ultimate goal is not consensus for its own sake but to ensure that variation reflects evidence and patient-centered outcomes rather than entrenched bias.

Clinically, a Bayesian approach integrates naturally with shared decision-making. Instead of framing results in binary terms (“this works” or “this does not”), shoulder surgeons can share their current probability estimates with the patient, including uncertainty. For example: “based on 10 studies, I estimate there is about a 75%-85% chance aTSA is more likely to match your preferences and values.” This makes it clear when decisions are being made under uncertainty rather than false certainty. Discussing uncertainty and probability also invites the patient to weigh outcomes most important to them, whether that's pain alleviation, motion, or revision risk. Decision aids could make this even more tangible by anticipating and reorienting common misconceptions and displaying probability curves and individualized potential harm and benefit estimates side by side, helping patients choose the option that best aligns with their values.

It is important to emphasize that this model is purely hypothetical. The choice of rTSA-favoring evidence was arbitrary, intended solely to illustrate the dynamics of belief change under Bayesian principles. No inference should be drawn regarding the superiority of rTSA over aTSA in actual clinical practice. The simulation used 10 rTSA-favoring trials solely for illustrative purposes. If the sequence of evidence had instead included balanced or contradictory findings (eg, 5 aTSA-favoring and 5 rTSA-favoring trials), belief updating trajectories would likely have diverged differently. The same simulation framework could be applied to any comparative surgical question, regardless of specialty or procedural focus. Finally, the simplicity of the model is both a strength and a limitation. By controlling for trial quality, publication bias, and heterogeneity, the simulation isolates the impact of prior beliefs and bias. However, real-world decision-making occurs in a more complex environment, where additional factors such as patient preferences, surgical logistics, and economic incentives may impact practice patterns. Future research could extend this model by incorporating these variables, calibrating bias strength using empirical data from surveys or decision-tracking studies, and validating predictions against observed trends in surgical registries.

## Conclusion

This Bayesian simulation demonstrated that prior beliefs and cognitive bias can markedly influence how shoulder surgeons interpret and act upon new evidence, even when that evidence is consistent and of high quality. This model demonstrates a phenomenon widely appreciated in principle—that biases shape interpretation of evidence—but does so in a structured, visual framework that may help clinicians and trainees concretely visualize belief dynamics.

## Disclaimers:

Funding: No funding was disclosed by the authors.

Conflicts of interest: Mariano E. Menendez, MD discloses Editorial Board Member of JSES; Consulting: Stryker/Tornier. David Ring, MD, PhD discloses IP Royalties, Patient +; editorial board, Clinical Orthopedics and Related Research: American Society for Surgery of the Hand, Chair Quality, Access, and Metrics Committee; board of directors, Orthopedic Trauma Association--Mental Health Task Force. Surena Namdari, MD, MSc discloses, research funding: DePuy, Integra LifeScience, OrthoFix, Wright Medical, Arthrex, Flexion Therapeutics, Biedermann Motech, OREF, DJO Surgical, Zimmer Biomet, Exactech, Roche; consulting: DJO Surgical, Biedermann Motech, Depuy Synthes, InGeneron, Vivo Capital; royalties: DJO Surgical, Biedermann Motech, Aevumed, Elsevier, SLACK, Wolters Kluwer; intellectual property: Biedermann Motech, DJO Surgical, Aevumed; ownership/stock: Parvizi Surgical Innovations, MD Live, MD Valuate, Force Therapeutics, HealthEXL, Aevumed, Tangen, Orthophor, RubiconMD, Rothman Orthopaedic Institute, OBERD; board member/advisor: Biedermann Lab for Orthopaedic Research, Aevumed, HealthEX, Philadelphia Orthopaedic Society. The other authors, their immediate families, and any research foundation with which they are affiliated have not received any financial payments or other benefits from any commercial entity related to the subject of this article.
